# Synthesis of Novel Farnesoid X Receptor Agonists and Validation of Their Efficacy in Activating Differentiation of Mouse Bone Marrow-Derived Mesenchymal Stem Cells into Osteoblasts

**DOI:** 10.3390/molecules24224155

**Published:** 2019-11-16

**Authors:** Ko Fujimori, Yusuke Iguchi, Yukiko Yamashita, Keigo Gohda, Naoki Teno

**Affiliations:** 1 Department of Pathobiochemistry, Osaka University of Pharmaceutical Sciences, 4-20-1 Nasahara, Takatsuki, Osaka 569-1094, Japan; 2 Faculty of Pharmaceutical Sciences, Hiroshima International University, 5-1-1, Hirokoshingai, Kure, Hiroshima 737-0112, Japan; y-iguchi@hirokoku-u.ac.jp (Y.I.); y-yamasi@hirokoku-u.ac.jp (Y.Y.); 3 Computer-aided Molecular Modeling Research Center, Kansai (CAMM-Kansai), 3-32-302, Tsuto-Otsuka, Nishinomiya, Hyogo 663-8241, Japan; ke.gohda@camm-kansai.org; 4 Graduate School of Pharmaceutical Sciences, Hiroshima International University, 5-1-1, Hirokoshingai, Kure, Hiroshima 737-0112, Japan; n-teno@hirokoku-u.ac.jp; 5 Faculty of Clinical Nutrition, Hiroshima International University, 5-1-1, Hirokoshingai, Kure, Hiroshima 737-0112, Japan

**Keywords:** mesenchymal stem cells, FXR, agonist, osteoblast, bone

## Abstract

The modulators of farnesoid X receptor (FXR), a bile acid receptor, regulate various biological processes including bile acid metabolism, and are associated with the control of fatty liver and osteoporosis. Thus, the control of FXR activity and development of FXR modulators are critical not only for research, but also for clinical application. In this study, we synthesized novel FXR agonists **1**–**4** possessing isoxazole and *N*-substituted benzimidazole moieties, and compared their effects on osteoblast differentiation with the known FXR agonists, chenodeoxycholic acid and a synthetic compound, GW4064. Two (**3** and **4**) of the four novel FXR agonists **1**–**4** showed high specificities for FXR. Computer-assisted modeling suggested that the binding of the FXR agonist **3** with ligand binding domain of FXR was similar to GW4064. FXR was expressed in mouse bone marrow-derived mesenchymal stem cell (MSC)-like ST2 cells (ST-2 MSCs). The FXR agonists activated the BMP-2-induced differentiation of ST-2 MSCs into osteoblasts and enhanced the expression of RUNX2. Moreover, the potency of the FXR agonist **3** was comparable to GW4064 in promoting osteoblast differentiation of ST-2 MSCs. These results indicate that FXR activation enhanced the BMP-2-induced differentiation of MSCs into osteoblasts through activating RUNX2 expression. FXR could be a potential therapeutic target for the treatment of bone diseases such as osteoporosis.

## 1. Introduction

Bone formation is a developmental process involving the differentiation of mesenchymal stem cells (MSCs) into osteoblasts [1]. Most of the bones are formed through endochondral ossification by the condensation of MSCs. The mouse bone marrow-derived MSC-like ST-2 cells (ST-2 MSCs) were established from the bone marrow of long bones of BC8 mice [2]. These multipotent cells can be differentiated into a variety of cells such as osteoblasts, chondrocytes, and adipocytes [3,4,5]. Osteoblast differentiation is tightly regulated by various factors such as hormones, transcription factors, and environmental conditions. A number of hormones such as parathyroid hormone, glucocorticoid, growth hormone, sex hormones, tumor growth factor-β, prostaglandins, and bone morphogenetic proteins (BMPs) are involved in the process of bone remodeling [6]. BMP-2 strongly activates the differentiation of MSCs into osteoblasts, and thereby the bone formation [7]. Moreover, runt-related transcription factor 2 (RUNX2, also known as Cbfa1, PEBP2A1, and AML3) has been firstly identified as an osteogenic transcription factor [8,9]. It plays important roles in the induction of differentiation of MSCs into osteoblasts [10] by activating the expression of a variety of osteogenic proteins such as type I collagen A1 (COL1A1), osteocalcin (OCN), and alkaline phosphatase (ALP) [8,11,12,13]. It was reported that RUNX2-null mice lacked endochondral and membranous ossification and osteoblast differentiation [9,14]. 

Farnesoid X receptor (FXR: NR1H4) is a member of the nuclear receptor superfamily [15], and has been identified as a receptor for bile acids such as chenodeoxycholic acid (CDCA; Supplementary Figure S1) and cholic acid, which are synthesized from cholesterol [16,17,18]. FXR forms a heterodimer with retinoid X receptor (RXR), which enhances gene expression by binding to the FXR-responsive element, also known as the inverted repeat-1, in the promoter region of the FXR-target genes [19,20,21,22]. FXR regulates a variety of physiological processes such as the metabolism of bile acids, lipids, and glucoses [23,24]. In addition, bile acids enhance the differentiation of MSCs into osteoblasts though FXR [25,26]. The ablation of FXR gene resulted in a decrease in bone mineral density [27] and deletion of small heterodimer partner (SHP), an FXR-target gene, decreased the bone mass through repression of osteoblast differentiation [28]. Thus, the FXR signaling is associated with the regulation of osteoblast differentiation; however, the underlying regulation mechanism has not yet been fully understood.

Representative FXR agonists include GW4064 [29] (Supplementary Figure S1) and 6α-ethylchenodeoxycholic acid (6-ECDCA, also known as obeticholic acid) [30]. Moreover, FXR appears to be a promising target for the treatment of nonalcoholic steatohepatitis (NASH) [31,32]. 6-ECDCA, a synthetic CDCA analog, is about 100-fold more potent than CDCA, and was approved by the Food and Drug Administration for the treatment of the NASH patients with primary biliary cirrhosis [30]. Tropifexor, a highly potent nonsteroidal FXR agonist, is undergoing the clinical trials for the treatment of NASH [33]. In contrast, although GW4064 is the potent nonsteroidal FXR agonist [34], it has not been used clinically. Thus, FXR agonists are of interest not only for research, but also for clinical application. As the potential FXR agonists for clinical application are few, novel potent FXR agonists are needed. Non-steroidal FXR agonists with [33,35,36,37,38,39,40,41,42,43,44,45,46] or without [47,48,49,50,51] isoxazole derivative have been discovered. Many of the former share isoxazole derivative and the structure modified stilbene moiety of GW4064. Among them, we focused on FXR agonists with the fused aromatic moiety (e.g., *N*-nonsubstituted benzimidazole) as the alternative to the stilbene while maintaining the isoxazole derivative [39], because we have previously disclosed a novel chemotype for FXR antagonists possessing an N-substituted benzimidazole scaffold and the synthetic knowledge for the building blocks containing benzimidazole [52,53,54]. In the early phase of research process of the development of FXR agonists, therefore, we selected the *N*-substituted benzimidazole as the alternative to the stilbene from the viewpoint of feasibility of the synthesis development and tackled using the scaffold for FXR antagonists in the development of a new chemotype for FXR agonists. 

To date, efforts have been made to improve the potency and efficacy for pharmacological applications [55,56]. In this study, our effort is first to clarify whether *N*-substituted benzimidazole scaffold can be used as a pharmacophore for FXR agonists. In fact, we synthesized four derivatives possessing *N*-substituted benzimidazole and investigated the effect of FXR agonists such as CDCA, GW4064, and novel potential FXR agonists **1**–**4** in the regulation of osteoblast differentiation of ST-2 MSCs. Moreover, we also evaluated whether the cellular system reported here is useful to assess the biological potential of low-molecular weight nonsteroidal and steroidal FXR agonists.

## 2. Results

### 2.1. Synthesis of Novel FXR Agonists

**Scheme 1:**
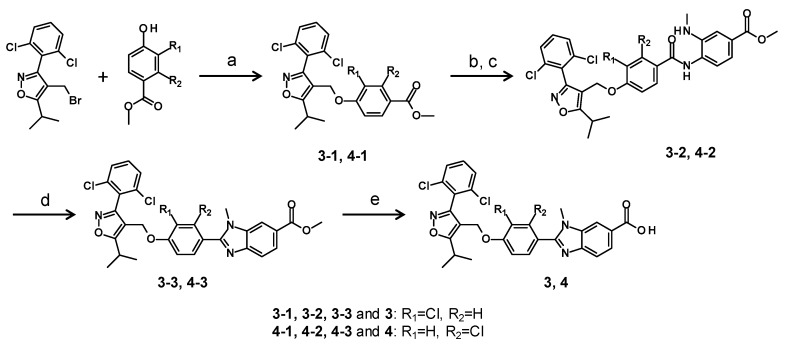
Synthetic pathway of novel FXR agonists **3** and **4**. Reagents and conditions: (a) K_2_CO_3_ and *N*,*N*-dimethylformamide (DMF) at 70 °C for 15 h; (b) 1M NaOH and methanol at 25 °C for 15 h; (c) methyl 4-amino-3-(methylamino) benzoate, 1-hydroxybenzotriazole (HOAt), 1-(3-dimethylaminopropyl)-3-ethylcarbodiimide hydrochloride (WSCI-HCl), and triethylamine at 0 to 25 °C for 15 h; (d) CH_3_COOH at 80 °C for 2 h; (e) 1M NaOH, methanol, and tetrahydrofuran (THF) at 25 °C for 15 h.

Different FXR modulators have some of the same shared structures; namely, the isoxazole moiety derived from GW4064 and the carboxylic acid group extending from the aromatic ring. The initial step in tackling FXR agonists was carried out using *N*-substituted benzimidazole with the carboxylic acid as the scaffold and the isoxazole moiety of GW4064. These two moieties were bridged by the aromatic derivatives to prepare the FXR agonists **1**–**4** (Scheme 1 and Supplementary Scheme S1).

The characterization of the novel FXR agonists was performed by ^1^H-NMR and high-resolution mass spectrometry (HRMS; Supplementary information). Their purities were confirmed by HPLC (Supplementary Table S1).

### 2.2. Time-Resolved Fluorescence Resonance Energy Transfer (TR-FRET) Binding Assay

An FXR TR-FRET binding assay was employed to characterize the binding abilities of FXR agonists **1**–**4** (Table 1). FXR agonist **1** possessing benzimidazole and isoxazole moieties bridged by phenyl ring appeared to bind nearly 2-fold greater than GW4064 (EC_50_: 43.7 ± 54.2 nM and 96.2 ± 62.7 nM, respectively). The EC_50_ value of FXR agonist **1** was modulated by the substitution of acidic moiety on benzimidazole such as FXR agonist **2**. Introduction of chloride to the phenyl ring (FXR agonists **3** and **4**) obviously showed differences in the results of TR-FRET binding assay. The potency of the substituent pattern of FXR agonist **3** was found to be high (EC_50_ = 13.4±7.9 nM, maximum activity 51.1 ± 27.6%; Table 1). 

**Figure 1:**
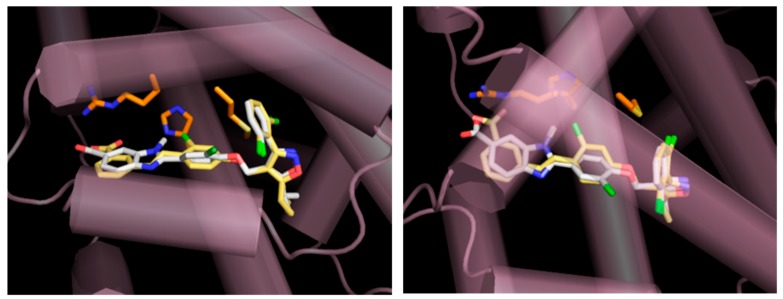
Modeling of FXR complexed with GW4064 and novel synthesized FXR agonist **3**. A complex-model of human FXRα-LBD monomer (PDB ID: 3DCT, purple cylinders and orange sticks) with GW4064 (X-ray; yellow sticks) and novel FXR agonist **3** (model; white sticks) was built using AutoDock Vina1.1.2. Left panel: view from the side. Right panel: view from above.

Additionally, computer-assisted modeling studies with the FXR agonist **3** were conducted to understand the binding mode of the FXR agonist **3** in the binding site of FXR ligand binding domain (LBD). The crystal structure of the LBD complexed with GW4064 was used for the modeling. Figure 1 showed that the FXR agonist **3** (white) occupied the same binding site of FXR LBD as GW4064 (yellow). The positions of the isoxazole moiety and the carboxyl group in the FXR agonist **3** were well overlapped to those of GW4064. In addition, the benzimidazole of the FXR agonist **3** and the stilbene of GW4064 have shared the same space in the LBD.

### 2.3. Specificity of FXR Agonists as Determined by Luciferase Reporter Assay

**Figure 2:**
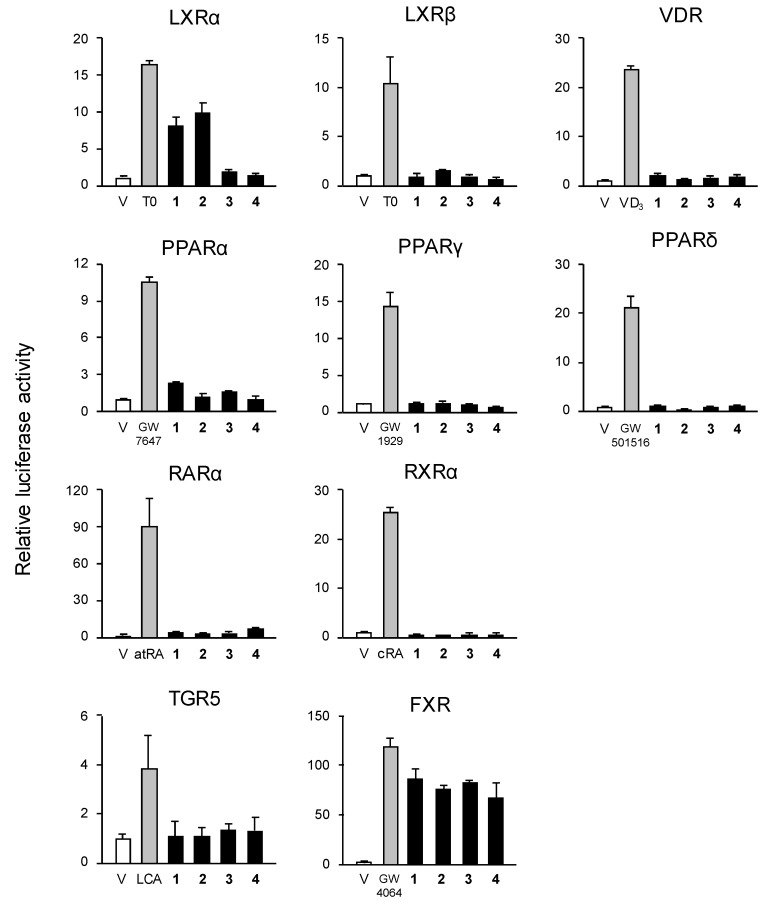
Specificity of FXR agonists to nuclear receptors. Huh-7, HEK293, or COS-7 cells (vehicle: V; white columns) were transfected with each of the plasmids for the nuclear receptor and the nuclear receptor-response element-driven luciferase gene. Then, the transfected cells were treated with each of the nuclear receptor agonists [LXRα/β: T0901317 (T0; 50 nM), VDR: 1α,25-vitamin D_3_ (VD_3_; 100 nM), PPARα: GW7467 (50 nM), PPARγ: GW1929 (50 nM), PPARδ: GW501516 (50 nM), RARα: all-trans-retinoic acid (atRA; 100 nM), RXRα: 9-cis-retinoic acid (cRA; 100 nM), TGR5: lithocholic acid (LCA; 200 nM), FXR: GW4064 (100 nM); gray columns] or novel FXR agonists **1**–**4** (1 μM; black columns). Luciferase activity was measured, as described in Materials and Methods. Data are shown as means ± S.D. from three experiments in triplicate assay.

The specificity of the novel FXR agonists against FXR was examined using various nuclear receptor response element-driven luciferase constructs. The novel FXR agonists **3** and **4** enhanced only FXR response element-driven luciferase activities among several nuclear receptors [liver X receptor (LXR) α and β, vitamin D receptor (VDR), peroxisome proliferator-activated receptor (PPAR) α, γ, and δ, retinoic acid receptor (RAR) α, RXRα, FXR, and transmembrane G protein-coupled receptor 5 (TGR5); Figure 2]. In contrast, the FXR agonists **1** and **2** increased both FXR- and LXRα-response element-driven luciferase activities (Figure 2). These results indicate that the novel FXR agonists **3** and **4** specifically activated FXR. Thus, we decided to use the novel FXR agonists **3** and **4** in further study.

### 2.4. Viability of *ST-2* MSCs

**Figure 3:**
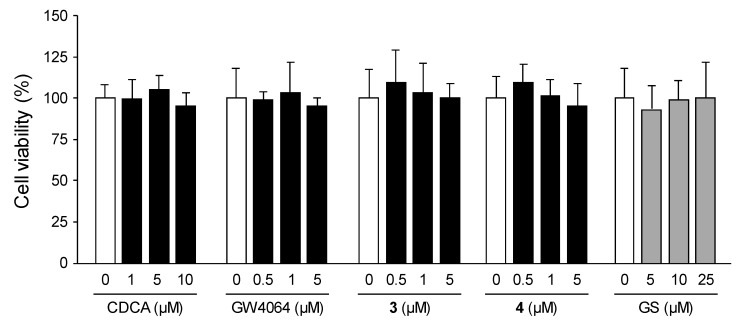
Cytotoxicity of FXR agonists and GS in ST-2 MSCs. The ST-2 MSCs (vehicle: V; white columns) were cultured for 12 days in the medium containing one of FXR agonists; 1–10 μM CDCA, 0.5–5 μM GW4064, 0.5–5 μM of novel FXR agonists **3** or **4** (black columns), and 5–25 μM GS (gray columns). Cell viability was measured by a WST assay. Data are shown as means ± S.D. from three experiments.

The cytotoxic effects of FXR agonists, CDCA, GW4064, and novel FXR agonists **3** and **4**, and an FXR antagonist (*Z*)-guggulsterone (GS) on ST-2 MSCs were determined by a WST assay. No cytotoxicity was observed after 12 days of incubation of ST-2 MSCs with any of the FXR agonists (Figure 3). Thus, we determined to use 10 μM CDCA, 5 μM GW4064, 5 μM of novel FXR agonist **3** and **4**, and 25 μM GS in the subsequent studies.

### 2.5. Expression of FXR and FXR-Target Genes during Osteoblast Differentiation of ST-2 MSCs

**Figure 4:**
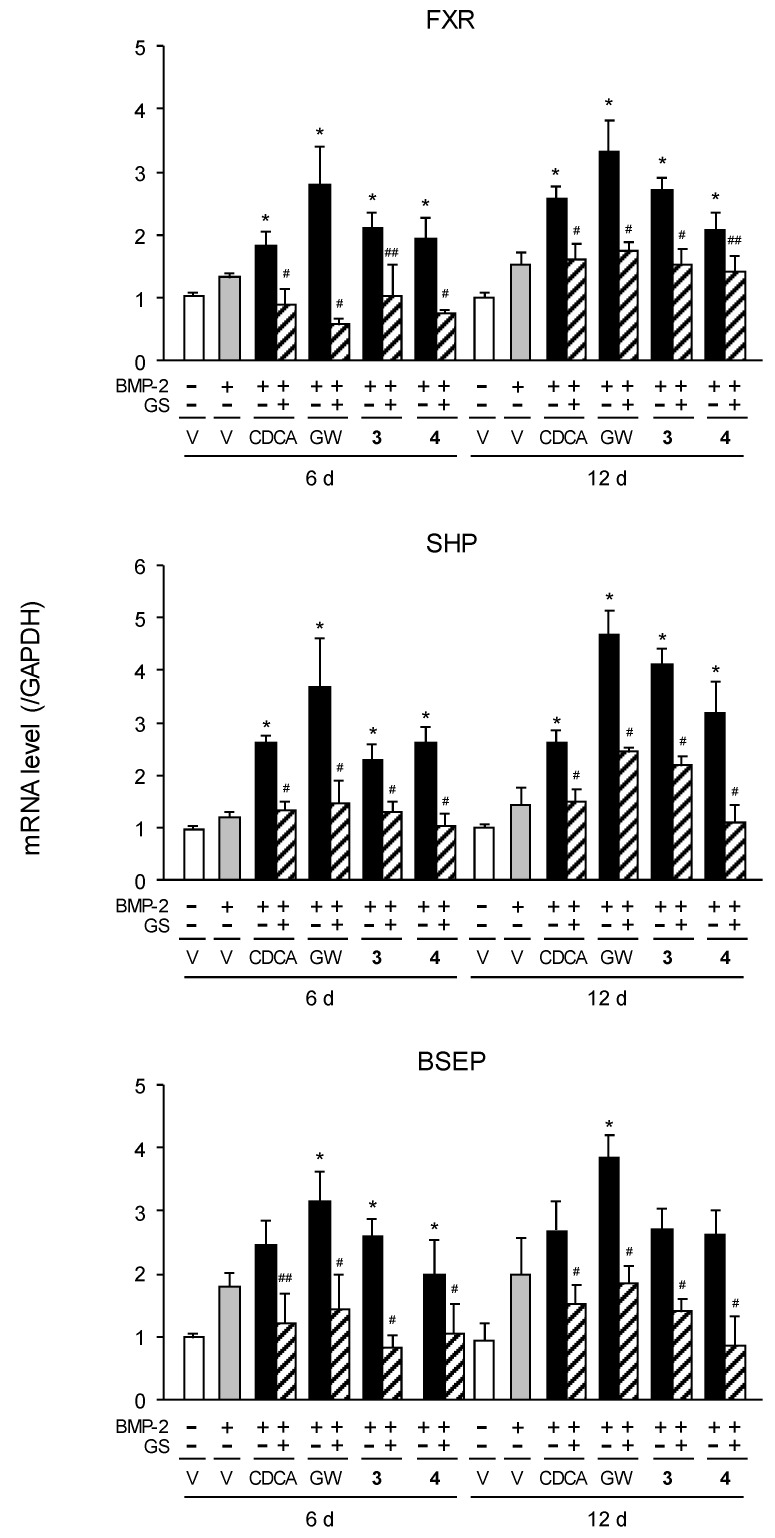
Expression of FXR and its target genes during osteoblast differentiation of ST-2 MSCs. The ST-2 MSCs (vehicle: V; white columns) were differentiated into osteoblasts for 6 or 12 days in the medium containing BMP-2 (50 ng/mL; gray columns) in the presence or absence of one of the FXR agonists, 10 μM CDCA, 5 μM GW4064 (GW), and 5 μM of novel synthesized FXR agonist **3** or **4** (black columns), and/or 25 μM GS (hatched columns). The expression levels were measured by quantitative PCR (qPCR). Data are presented as means ± S.D. from three experiments. * *p* < 0.01, as compared with the BMP-2-treated cells, ^#^ *p* < 0.01, ^##^ *p* < 0.05, as compared with the FXR agonist-treated cells.

We measured the expression levels of the FXR and FXR-target genes during osteoblast differentiation of ST-2 MSCs. The cells were differentiated into osteoblasts for 6 or 12 days in the presence of BMP-2 together with one of the FXR agonists and/or the FXR antagonist, GS. The expression of the FXR gene was induced by the treatment with BMP-2 at 6 and 12 days (Figure 4). The mRNA expression level of the FXR was elevated upon treatment with CDCA, GW4064, or the novel FXR agonists **3** and **4** at 6 and 12 days (Figure 4). The increase in gene expression caused by the FXR agonists was repressed by the co-treatment with GS (Figure 4). Furthermore, the expression of the FXR-target genes; bile salt export pump (BSEP, also known as ABCB11) and SHP, was also enhanced by the treatment with each of the FXR agonists. In contrast, the FXR agonist-induced expression was suppressed by co-treating with GS (Figure 4). These results indicate that the known and novel synthesized FXR agonists enhanced the expression of the FXR and its target genes during osteoblast differentiation of ST-2 MSCs.

### 2.6. Enhancement of ALP Activity by FXR Agonists during Osteoblast Differentiation of ST-2 MSCs

**Figure 5:**
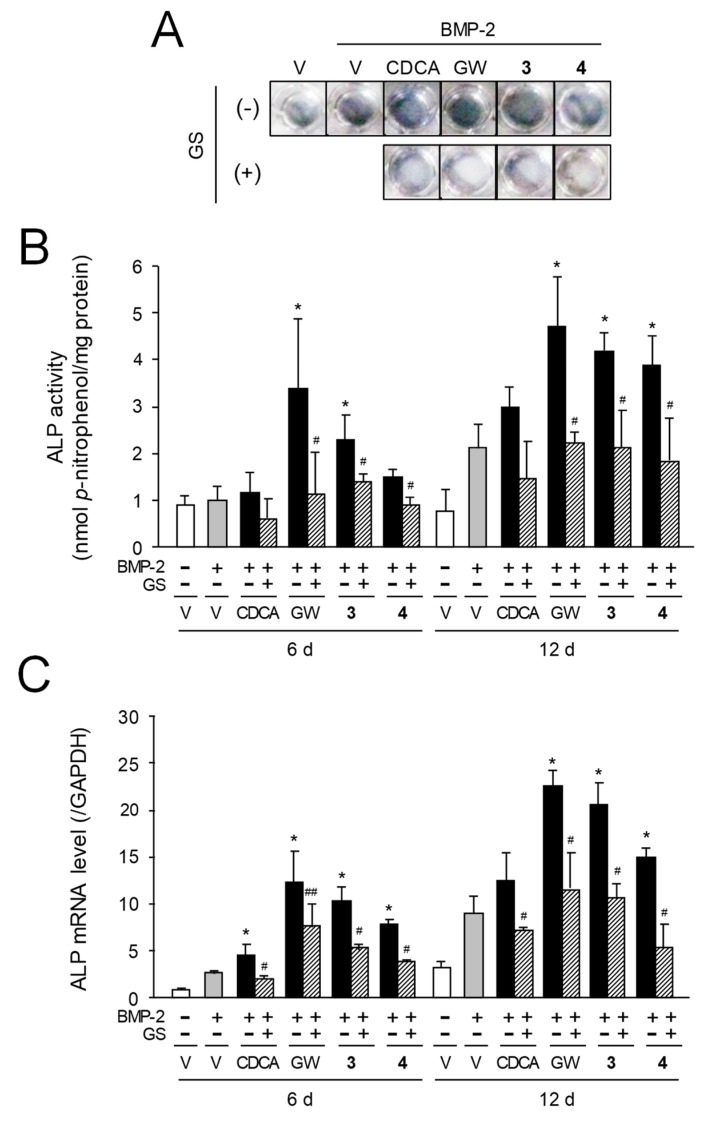
ALP activity in BMP-2-induced differentiation of ST-2 MSCs into osteoblasts. (**A**) ALP staining. ST-2 MSCs were differentiated into osteoblasts for 12 days in the medium containing BMP-2 (50 ng/mL) together with 10 μM CDCA, 5 μM GW4064 (GW), or 5 μM of novel synthesized FXR agonist **3** or **4** in the presence or absence of 25 μM GS. ALP staining was carried out as described in Materials and Methods. The results are the representative from three experiments. (**B**) ALP activity. The ST-2 MSCs (vehicle: V; white columns) were cultured for 6 or 12 days in the medium containing BMP-2 (50 ng/mL; gray columns) together with one of the FXR agonists, 10 μM CDCA, 5 μM GW4064 (GW), and 5 μM of novel synthesized FXR agonist **3** or **4** (black columns) in the presence or absence of 25 μM GS (hatched columns). Data are shown as means ± S.D. for three experiments. *p < 0.01, as compared with the BMP-2-treated cells, ^#^ *p* < 0.01, as compared with the FXR agonist-treated cells. (**C**) Expression of the ALP gene during osteoblast differentiation of ST-2 MSCs. The cells were cultured as described in the legend of Figure 5B. Expression level of the ALP gene was measured by qPCR. Data are presented as means ± S.D. from three experiments. * *p* < 0.01, as compared with the BMP-2-treated cells, ^#^ *p* < 0.01, ^##^ *p* < 0.05, as compared with the FXR agonist-treated cells.

The ST-2 MSCs were differentiated into osteoblasts for 6 or 12 days in RPMI-1640 medium with BMP-2 in the presence or absence of one of the FXR agonists and/or GS. The staining and activity of ALP were enhanced by treating with BMP-2 during osteoblast differentiation of ST-2 MSCs at 6 days (Figure 5A,B). The staining and activity of ALP were elevated by the treatment with CDCA and GW4064, as compared with that in the BMP-2-treated ST-2 MSCs at 6 days (Figure 5A,B). Moreover, when the cells were differentiated into osteoblasts for 12 days with BMP-2 and/or CDCA or GW4064, the ALP staining and activity were further increased (Figure 5A,B). The CDCA- and GW4064-induced staining and activity of ALP were suppressed by co-treating with GS (Figure 5A,B). Furthermore, the novel FXR agonists **3** and **4** enhanced the staining and activity of ALP (Figure 5A,B), and the ability of the FXR agonist **3** was comparable to that of GW4064 in the 6- or 12-days-differentiated ST-2 MSCs (Figure 5A,B). The enhancement of ALP staining and activity caused by the novel FXR agonists **3** and **4** was repressed by co-incubating with GS (Figure 5A,B).

We measured the expression level of the ALP gene in FXR agonist-treated ST-2 MSCs. The ALP mRNA level was enhanced by the treatment with BMP-2, as compared with that in the vehicle-treated ST-2 MSCs at 6 days (Figure 5C). When the cells were differentiated for 6 days with BMP-2 together with CDCA or GW4064, the transcription level of the ALP gene was elevated, as compared with that in the BMP-2-treated ST-2 MSCs (Figure 5C). Moreover, BMP-2-, CDCA-, and GW-4064-induced ALP gene expressions were further elevated at 12 days (Figure 5C). In addition, the FXR agonist-induced ALP gene expression was repressed by co-treating with GS (Figure 5C). The novel FXR agonists **3** and **4** could also enhance ALP gene expression (Figure 5C). Especially, the ability of the FXR agonist **3** was comparable to that of GW4064 in the ALP gene expression during osteoblast differentiation of ST-2 MSCs (Figure 5C). Furthermore, the enhancement of ALP gene expression by the novel FXR agonists **3** and **4** was suppressed by the co-treatment with GS (Figure 5C). These results, taken together, indicate that GW4064 and novel FXR agonists **3** and **4** activated the ALP activity through FXR in the BMP-2-induced osteoblast differentiation of ST-2 MSCs. Moreover, the novel FXR agonist **3** showed comparable potency to GW4064 in the promotion of ALP activity during osteoblast differentiation of ST-2 MSCs.

### 2.7. Elevation of the Expression of Osteogenic Genes by FXR Agonists in ST-2 MSCs

**Figure 6:**
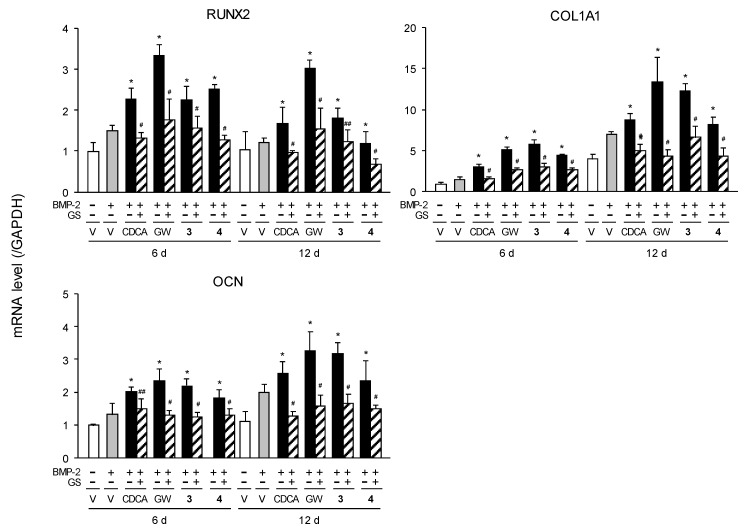
Expression of osteogenic genes during osteoblast differentiation of ST-2 MSCs. The ST-2 MSCs were differentiated into osteoblasts as described in the legend of Figure 5B. Transcription levels of the RUNX2, COL1A1, and OCN genes were measured by qPCR. Data represent as means ± S.D. from three experiments. * *p* < 0.01, as compared with the BMP-2-treated cells, ^#^ *p* < 0.01, ^##^ *p* < 0.05, as compared with the FXR agonist-treated cells.

Next, we measured the changes in the expression levels of the genes involved in the osteoblast differentiation of ST-2 MSCs. When the cells were differentiated into osteoblasts in the medium containing BMP-2, the expression level of the RUNX2, COL1A1, and OCN genes was all enhanced at 6 and 12 days after initiation of differentiation (Figure 6). CDCA and GW4064 further enhanced the expression of these genes at 6 days, as compared with those in the BMP-2-treated cells (Figure 6). Moreover, the novel FXR agonist **3** and **4** elevated the expression of these genes at 6 days, and the FXR agonist **3**-induced increase was comparable to that of GW4064 (Figure 6). Interestingly, the FXR-induced RUNX2 gene expression at 12 days was lower than that at 6 days, indicating that FXR activation is important for the enhancement of RUNX2 gene expression during the early phase of osteoblast differentiation of ST-2 MSCs (Figure 6). Additionally, the novel FXR agonist-induced expression of the osteogenic genes was repressed by co-treating with GS (Figure 6). These results reveal that the known and novel FXR agonists could activate the BMP-2-induced osteoblast differentiation of ST-2 MSCs via activating RUNX2 expression. It is noteworthy that the novel FXR agonist **3** showed high potency, which is comparable to that of GW4064 in osteoblast differentiation of ST-2 MSCs.

## 3. Discussion

A variety of FXR modulators have been discovered and are classified as steroidal or non-steroidal, based on their chemical structures [57]. Although FXR is associated with the regulation of various biological processes and diseases [23,24,31,32], during the validation of FXR modulators for drug development, most compounds dropped out in the preclinical and clinical stages and only a few could enter the clinical trials [57].

Benzimidazole derivatives are generally useful intermediates/subunits in the development of molecules of pharmaceutical or biological interest. Substituted benzimidazole derivatives have found applications in diverse therapeutic areas [58]. The synthetic protocols have also been reported to prepare many benzimidazole derivatives through the variation of commercially available starting materials [59,60,61]. FXR agonists previously reported by Akwabi-Ameyaw et al. have the fused aromatic rings (e.g., benzothiophene or *N*-nonsubstituted benzimidazole) as the alternative to the stilbene of GW4064 and their compounds reveal the agonistic activity against FXR at nanomolar levels [39]. Encouraged by the observation obtained from the previous report [39], and considering the stability of the derivatives such as benzothiophene (e.g., labile for oxidation) [62] and a wide diversity of the building blocks containing *N*-substituted benzimidazole moiety [52,53,54], exploring a new series of FXR agonists was initiated. We synthesized four compounds (**1**–**4**) to verify the usefulness of the moiety as FXR agonists and determined their potential effects in osteoblast differentiation of ST-2 MSCs. Even the distinct of small modification on these FXR agonists (e.g., **1** vs. **2**, **1** vs. **3**, **1** vs. **4**, and **3** vs. **4**) reflects to the EC_50_ values of TR-FRET binding assay (Table 1). Eventually, FXR agonist **3** having *N*-substituted benzimidazole as the pharmacophore was observed to have higher binding activity than GW4064 (Table 1). From the modeling results, the FXR agonist **3** occupied the same binding space of GW4064. The binding space of FXR is known as hydrophobic circumstances [63]. Since the FXR agonist **3** has the hydrophobic *N*-substituted benzimidazole, the FXR agonist **3** would show higher binding activity than GW4064. The FXR agonist **3** showed high potency in FXR-mediated BMP-2-induced osteoblast differentiation (Figure 5 and Figure 6). Thus, owing to its biological properties, the FXR agonist **3** could be a lead compound for developing a chemotype that is different from the existing FXR agonists. Furthermore, the results obtained from the modeling study implied that there is the vacant space of the LBD that is not occupied by GW4064 (Figure 1). As a clue for further molecular modifications on the basis of the *N*-substituted benzimidazole moiety, the space could be filled with a modification of FXR agonist **3**, especially a building block extended from benzimidazole. In addition, we validated another FXR agonists **1**, **2**, and **4** in this study. Novel FXR agonists **1** and **2** activated through FXR and LXRα in the luciferase assay (Figure 2). While, FXR agonists **3** and **4** have specificities to FXR and activated the osteoblast differentiation of ST-2 MSCs. The potency of FXR agonist **3** is higher than FXR agonist **4** and comparable to GW4064 in BMP-2-induced osteoblast differentiation of ST-2 MSCs. Therefore, we could develop a new chemotype for FXR agonists with increased agonistic activity.

Osteoblast differentiation is regulated via complex and multiple steps by various factors [13,64]. Nuclear receptors are ligand-dependent transcription factors that regulate various diseases such as diabetes, cardiovascular disease, obesity, and osteoporosis [65]. Some nuclear receptors are involved in the control of bone formation and osteoblast differentiation [65]. It is known that the activation of FXR induces osteoblast differentiation [25,26]. Bile acids, ligands for FXR, activate the differentiation of MSCs into osteoblasts [25,26]. FXR gene-deficient mice were reported to have lower bone mineral density [27]. However, the molecular mechanism of FXR-mediated osteoblast differentiation remains unclear. In this study, we investigated the role of FXR in osteoblast differentiation of ST-2 MSCs using the known and novel FXR agonists. CDCA is a bile acid derived from cholesterol that has been recognized as a natural ligand of FXR [16]. In addition, a synthetic compound, GW4064 has been shown to be the most potent FXR agonist [34]. Importantly, the novel FXR agonist **3** showed similar effects as GW4064 in the promotion of BMP-2-induced differentiation of ST-2 MSCs into osteoblasts (Figure 5 and Figure 6). The role of FXR in osteoblast differentiation of ST-2 MSCs was further demonstrated using the FXR antagonist GS, which clearly decreased the FXR agonist-promoted osteoblast differentiation of ST-2 MSCs. Moreover, we further have to prove the specificity of novel FXR agonists against FXR using FXR-deficient cells.

Bone formation (osteoblastogenesis) is coupled to bone resorption (osteoclastogenesis) in the bone homeostasis (bone remodeling and repair). An imbalance in bone homeostasis results in diseases such as osteoporosis and osteopenia [66]. We observed that FXR activation enhanced osteoblast differentiation (Figure 5 and Figure 6), whose results are supported by the previous finding that bile acid promoted osteoblastogenesis through FXR [26,27]. Moreover, bone mineral density was decreased [27] and osteoclast differentiation was enhanced in FXR gene-knockout mice [67]. Therefore, FXR is critical in the regulation of not only bone formation (osteoblastogenesis), but also bone resorption (osteoclastogenesis). Osteoblast differentiation of ST-2 MSCs was induced by the endogenous FXR ligand, a bile acid CDCA (Figure 5 and Figure 6), indicating that bile acid is associated with the bone formation [27]. In addition, bile acids suppressed osteoclast differentiation [67]. Thus, bone remodeling might be regulated by bile acids, indicating that bone homeostasis could be associated with liver activity. The association between bone remodeling and the liver activity through FXR should be further investigated using known and novel FXR modulators.

Another important finding in the present study is that FXR activation enhanced the expression of RUNX2 in the early stage of BMP-2-induced differentiation of ST-2 MSCs into osteoblasts (Figure 6). RUNX2 is a key protein in the activation of osteoblast differentiation and plays an important role in the commitment of BMP-2-induced osteoblast differentiation from mouse C2C12 myoblasts [68,69]. A variety of transcription factors are involved in the transcriptional regulation of the RUNX2 gene during osteoblast differentiation [70]. The homeodomain proteins such as distal-less homeobox (DLX) 3, DLX5, and Msh homeobox 2 (MSX2) are important in the regulation of the early stage of osteoblast differentiation of MSCs [71]. However, the expression level of these homeodomain proteins was not affected by FXR agonists in the BMP-2-induced differentiation of ST-2 MSCs into osteoblasts (data not shown). From another perspective, FXR activation may stimulate the BMP-2 signaling in the elevation of the RUNX2 expression during osteoblast differentiation of MSCs, because the BMP-2 signaling stimulated the RUNX2 expression in osteoblast differentiation of MSCs [72]. The mechanism of transcriptional activation of the RUNX2 gene by FXR in the promotion of the early stage of osteoblast differentiation of MSCs should be further investigated.

## 4. Materials and Methods 

### 4.1. Materials

GW4064, (Z)-guggulsterone (GS), T0901317, 1α,25-vitamin D_3_, GW7647, and GW1929 were obtained from Cayman Chemical (Ann Arbor, MI, USA). CDCA, lithocholic acid, and Fast-blue BB salt were purchased from Sigma (St. Louis, MO, USA). GW501516 was from ChemScene LLC (Newark, NJ, USA). All-trans retinoic acid and 9-cis-retinoic acid were from FUJIFILM Wako Pure Chemical (Osaka, Japan). BMP-2 was from Miltenyi Biotec. (Bergisch Gladbach, Germany). Naphthol AS-MX phosphate was from Nacalai Tesque (Kyoto, Japan). Amino acid derivative and the coupling reagents were from Watanabe Chemical (Hiroshima, Japan) or Peptide Institute (Osaka, Japan). All other chemicals were from Tokyo Chemical Industry (Tokyo, Japan), FUJIFILM Wako Pure Chemical, and Aurora Fine Chemicals (San Diego, CA, USA) unless otherwise stated.

### 4.2. Synthesis of FXR Agonists

The synthetic schemes for novel FXR agonists **1**–**4** were depicted in Supplementry Scheme S1 (**1**, **2**) and Scheme 1 (**3**, **4**). The isoxazole moiety of GW4064 [34] and *N*-substituted benzimidazole part [53,54] were prepared according to the previous reports. The isoxazole derivative was coupled with 4-hydroxybenzoate derivatives in DMF containing K_2_CO_3_ to yield **3**–**1** and **4**–**1**. After removal of the methyl ester group of **3**-**1** and **4**-**1** (Scheme 1), subsequent coupling with methyl 4-amino-3-(methylamino)benzoate [52] using HOAt, WSCI-HCl, and triethylamine in DMF yielded **3**–**2** and **4**–**2**. Immediate ring closure of **3**–**2** and **4**–**2** in CH_3_COOH gave the corresponding N-substituted benzimidazole derivatives, **3**–**3** and **4**–**3**. Removal of methyl esters of **3**–**3** and **4**–**3** was achieved by 1 M NaOH in methanol/THF to give **3** and **4**. 

### 4.3. TR-FRET Binding Assay

The binding activity of FXR agonists was determined using a LanthaScreen TR-FRET FXR Coactivator Assay Kit (Thermo Fisher Scientific, Waltham, MA, USA) as described previously [53]. TR-FRET signal was detected by an EnVision Multilabel Plate Reader (Perkin Elmer, Waltham, MA, USA). The EC_50_ values of each compound were calculated using a probit analysis [53]. Maximum activities (%) were determined by calculating the percentage activities with respect to that of 0.5 μM of GW4064 [73].

### 4.4. Modeling of FXR Complex with GW4064 and Novel FXR Agonist **3**

Model complexes of the LBD of human FXRα with GW4064 (PDB ID: 3DCT) and novel FXR agonist **3** were built using AutoDock Vina1.1.2 with the standard parameters [74].

### 4.5. Luciferase Reporter Assay

Luciferase reporter assay was performed as described previously [53]. Briefly, human hepatoma Huh-7 (for FXR, RARα, and RXRα), transformed African green monkey kidney fibroblast COS-7 (for LXRα/β and PPARα/γ/δ), or human embryonic kidney HEK293 (for VDR and TGR5) cells were transfected with each of plasmids for nuclear receptor and nuclear receptor-response element-driven luciferase gene. Then they were treated with each of nuclear receptor agonists [LXRα/β: T0901317 (T0; 50 nM), VDR: 1α,25-vitamin D_3_ (VD_3_; 100 nM), PPARα: GW7467 (50 nM), PPARγ: GW1929 (50 nM), PPARδ: GW501516 (50 nM), RARα: all-trans-retinoic acid (atRA; 100 nM), RXRα: 9-cis-retinoic acid (cRA; 100 nM), bile acid-activated receptor (TGR5): lithocholic acid (LCA; 200 nM), FXR: GW4064 (100 nM)] or novel FXR agonists **1**–**4** (1 μM). The luciferase activity was measured as described previously [53].

### 4.6. Cell Culture

Mouse ST-2 MSCs were obtained from RIKEN BioResource Research Center (Tsukuba, Japan) and cultured in RPMI-1640 medium (Sigma) containing 10% (*v*/*v*) fetal calf serum and antibiotics (100 units/mL penicillin and 100 μg/mL streptomycin; Nacalai Tesque) at 37 °C in an atmosphere of 5% CO_2_. The ST-2 MSCs were allowed to differentiate into osteoblasts for 6 or 12 days in RPMI-1640 medium with BMP-2 (50 ng/mL). The medium was changed every 3 days.

### 4.7. Cell Toxicity Assay

The ST-2 MSCs were cultured in a 96-well plate in RPMI-1640 medium containing one of various concentrations of FXR agonists (0–10 μM CDCA, 0–5 μM GW4064, and 0–5 μM of novel FXR agonists **3** or **4** or 0–25 μM GS) for 12 days. The cells were washed twice with PBS, and cell viability was determined using Cell Counting Kit-8 (Dojindo Molecular Technologies, Kumamoto, Japan) according to the protocols provided by the supplier. The cell viability (%) was shown relative to the vehicle-treated cells (0 μM).

### 4.8. RNA Extraction and Quantitative PCR 

RNAiso Plus was used to isolate total RNA (Takara-Bio, Kyoto, Japan). Subsequently total RNA (1 μg) was reverse-transcribed to cDNAs using ReverTra Ace reverse transcriptase (Toyobo, Osaka, Japan) and random hexamer (Takara-Bio). Thereafter, the mRNA levels were determined by qPCR analysis using a Power SYBR Green Master Mix (Thermo Fisher Scientific) in an Applied Biosystems 7500 Real Time PCR System (Thermo Fisher Scientific). The sequences of the gene-specific primers were shown in Table 2. The relative mRNA expression levels were determined using the 2^−ΔΔCT^ method and normalized to that of glyceraldehyde-3-phosphate dehydrogenase (GAPDH).

### 4.9. ALP Staining

The ST-2 MSCs were allowed to differentiate into osteoblasts for 12 days in RPMI-1640 medium with BMP-2 (50 ng/mL) and one of FXR agonists together with or without GS (25 μM). Then, the cells were washed twice with PBS and fixed with 10% (*v*/*v*) formaldehyde in PBS at 25 °C for 10 min. The cells were again washed with PBS, and incubated with the staining solution (0.1 mg/mL naphthol AS-MX phosphate and 0.6 mg/mL Fast-blue BB salt in 0.1 M Tris-Cl, pH 8.8) at 25 °C for 30 min. After removing the staining solution, the cells were washed with PBS and visualized under a microscope (CKX-41, Olympus, Tokyo, Japan).

### 4.10. ALP Activity Assay

The ST-2 MSCs were cultured in a 96-well plate for 6 or 12 days in RPMI-1640 medium containing BMP-2 (50 ng/mL) together with one of the FXR agonists [**3** or **4** (5 μM), CDCA (10 μM), or GW4064 (1 μM)] and/or GS (25 μM). The ALP activity was measured using LabAssay ALP (FUJIFILM Wako Pure Chemical) according to the manufacturer’s protocols. Protein concentrations were measured using a Pierce BCA Protein Assay Kit (Thermo Fisher Scientific).

### 4.11. Statistical Analysis

Data are expressed as the means ± S.D. Statistical analysis was performed using an unpaired *t* test or one-way ANOVA. Tukey’s post hoc test was used for multiple comparisons. A value of *p* < 0.05 was considered statistically significant.

## 5. Conclusions

Novel FXR agonists **3** and **4**, possessing isoxazole and *N*-substituted benzimidazole moieties showed high specificities to FXR. Computer-assisted modeling suggested that FXR agonist **3** occupied the same binding site of FXR LBD as GW4064. FXR activation promoted the early stage of BMP-2-induced osteoblast differentiation of ST-2 MSCs through activation of RUNX2, indicating that FXR is a potential target for developing the medicines for the treatment of bone diseases. Moreover, the FXR agonist **3** has high potency, which is comparable to the synthetic FXR agonist, GW4064, in the promotion of osteoblast differentiation. Additionally, the cellular system employed in this study is useful for validating the abilities of FXR modulators and for elucidating the FXR-mediated regulatory mechanism.

## Figures and Tables

**Table 1:** Binding ability of FXR agonists by TR-FRET binding assay.

FXR Agonist	EC_50_ (nM)	Maximum Activity (%)
GW4064	96.2 ± 62.7	135.8 ± 42.6
**1**	43.7 ± 54.2	58.2 ± 26.2
**2**	64.4 ± 135.1	58.3 ± 20.4
**3**	13.4 ± 7.9	51.1 ± 27.6
**4**	31.6 ± 26.9	76.4 ± 23.4

**Table 2:** Nucleotide sequences of primers used in qPCR.

Gene	Accession No.	Forward Primer	Reverse Primer
*FXR*	NM_001163700	5′-GGAGAGGAAGACTCAGTCCAGA-3′	5′-GCCTGGACGACAGAGCTAAA-3′
*BSEP*	NM_021022	5′-GGACACACCTGAGAGGACCTT-3′	5′-AGATCGTTGACGGATGGAAG-3′
*SHP*	NM_011850	5′-CTGAAGGGCACGATCCTCT-3′	5′-GCCTCCTGTTGCAGGTGT-3′
*ALP*	NM_007431	5′-AACCCAGACACAAGCATTCC-3′	5′-GAGAGCGAAGGGTCAGTCAG-3′
*RUNX2*	NM_009820	5′-GCACCGACAGTCCCAACT-3′	5′-CTCCGAGGGCTACAACCTT -3′
*COL1A1*	NM_007742	5′-CATGTTCAGCTTTGTGGACCT-3′	5′-GCAGCTGACTTCAGGGATGT-3′
*OCN*	NM_001032298	5′-AGACTCCGGCGCTACCTT-3′	5′-CTCGTCACAAGCAGGGTTAAG-3′
*GAPDH*	NM_008084	5′-CAAGGAGTAAGAAACCCTGGACC-3′	5′-CGAGTTGGGATAGGGCCTCT-3′

## Supplementary Materials

The following are available online: Figure S1: Chemical structure of potent FXR agonists, Scheme S1: Synthetic pathway of novel FXR agonists 1 and 2., Supplementary material: Identification of FXR agonists by mass spectroscopy and NMR spectrum., Table S1: Purity of novel synthetic FXR agonists.
