# Supplementary material for: Synthesis of Novel Farnesoid X Receptor Agonists and Validation of Their Efficacy in Activating Differentiation of Mouse Bone Marrow-Derived Mesenchymal Stem Cells into Osteoblasts
Source: Molecules. 2019 Nov 16;24(22):4155. doi: 10.3390/molecules24224155 (PMC6891315; doi:10.3390/molecules24224155)
Supplement: Supplementary file 1 [file molecules-24-04155-s001.pdf]

molecules

MDPI

Supplemental data

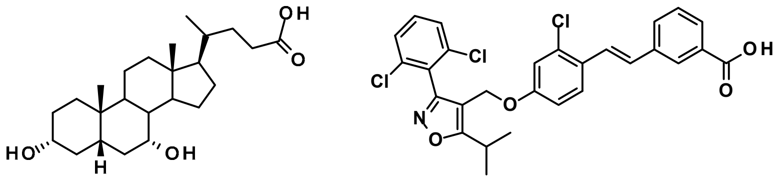

CDCA GW4064

Figure S1. Chemical structure of potent FXR agonists

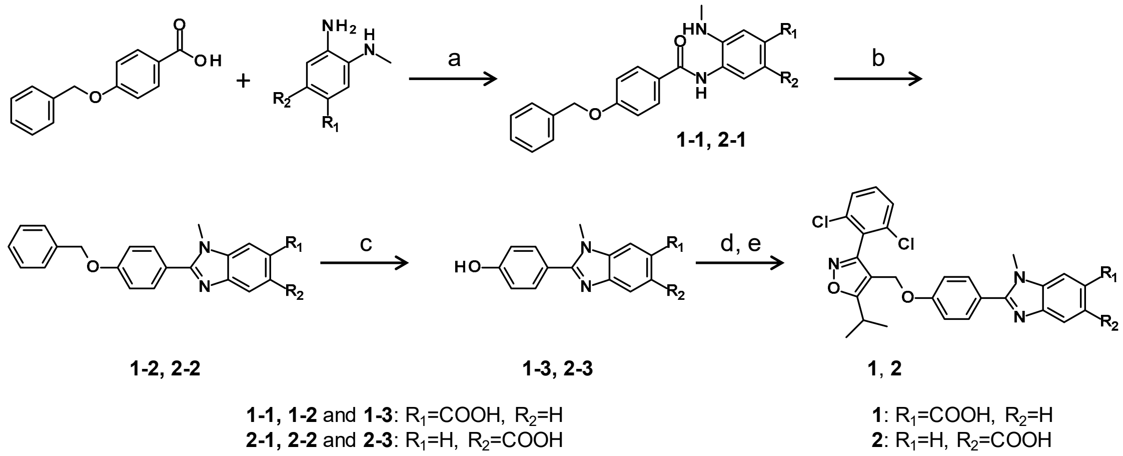

Scheme S1. Synthetic pathway of novel FXR agonists 1 and 2.

Reagents and conditions: (a) HOAt, WSCI-HCl, DMF, at 0 °C to 20 °C for 15 h; (b) CH_3COOH at 80 °C for 5 h; (c) 10% palladium on carbon (Pd/C), H_2, and methanol at 20 °C for 15 h; (d) 4-(chloromethyl)-3-(2,6-dichlorophenyl)-5-isopropyl-isoxazole, K_2CO_3, and DMF at 70 °C for 15 h; (e) 1 M NaOH, methanol, and THF at 20 °C for 30 h.

Identification of FXR agonists by mass spectroscopy and NMR spectrum

^{1}H-NMR experiments were performed using a JMTC-600 600 NMR spectrometer (JEOL; Tokyo, Japan) with $CDCl_3$ or DMSO-d_6 as solvents. Chemical shifts were expressed in parts per million (ppm, δ) and referred to the solvent signal. High-resolution mass spectra were recorded on the JMS-T100LC AccuTOF equipped with an electrospray ion source (JEOL). The analytical HPLC system consisted of CBM-20A System Controller, LC-20AT Pump Unit, DGU-20A3R In-Line Degasser, SPD-20A Absorbance Detector, FCV-11AL Valve Unit, and FRC-10A Fraction Collector (SHIMADZU, Kyoto, Japan). The absorbance detector was operated at 254 nm. The mobile phase for analysis was a combination of water (A) and acetonitrile (B), both containing 0.1%(v/v) TFA, and the flow rate was 1 ml/min. TSK gel ODS column (4.6 x 150 mm for analysis; Tosoh, Tokyo, Japan) was used.

Synthetic protocol of 1:

Methyl 4-[(4-benzyloxybenzoyl)amino]-3-(methylamino)benzoate (1–1):

Methyl 4-amino-3-(methylamino)benzoate (0.51 g, 2.83 mmol) in DMF (3 ml) was added to a solution of 4-benzyloxybenzoic acid (0.64 g, 2.83 mmol) in DMF (2 ml) at 0 °C. Subsequently, HOAt (0.41 g, 3.1 mmol) and WSCI-HCl (0.59 g, 3.1 mmol) were added to the former solution at 0 °C and stirred at 20 °C for 15 h. The reaction mixture was quenched with sat. NH_4Cl and extracted with CH_2Cl_2. The combined extracts were washed with H_2O and brine, dried over $MgSO_4$, and concentrated under reduced pressure. The residue was purified by the silica gel column chromatography to give **1–1** in 25% yield. Rf = 0.60 [*n*-hexane/ethyl acetate (ethyl acetate) = 1/1], ^{1}H NMR (600 MHz, DMSO-d_6): δ 9.61 (s, 1H), 8.02 (d, J = 8.6 Hz, 2H), 7.52 (d, J = 7.4 Hz, 2H), 7.46 (t, J = 7.4 Hz, 2H), 7.41 (t, J = 7.9 Hz, 2H), 7.30 (d, J = 8.0 Hz, 1H), 7.21 (s, 1H), 7.18 (d, J = 8.7 Hz, 2H), 5.56 (brs, 1H), 5.27 (s, 2H), 3.89 (s, 3H), 2.81 (d, J = 4.8 Hz, 3H).

Methyl 2-(4-benzyloxyphenyl)-3-methyl-benzimidazole-5-carboxylate (1–2):

1–1 (270 mg, 0.69 mmol) was dissolved in CH_3COOH (10 ml) at 20 °C and stirred at 80 °C for 5 h. The reaction mixture was evaporated and quenched by addition of sat. $NaHCO_3$, followed by extracted with ethyl acetate. Organic layer was washed successively with H_2O and brine, dried over $MgSO_4$, and concentrated under reduced pressure. The residue was purified by the silica gel column chromatography to give **1–2** in 98% yield. Rf = 0.55 (*n*-hexane/ethyl acetate = 1/1), ^{1}H NMR (600 MHz, DMSO-d_6): δ 8.28 (s, 1H), 7.92 (d, J = 8.4 Hz, 1H), 7.90 (d, J = 8.6 Hz, 2H), 7.79 (d, J = 8.4 Hz, 1H), 7.55 (d, J = 7.4 Hz, 2H), 7.48 (t, J = 7.5 Hz, 2H), 7.41 (t, J = 7.3 Hz, 1H), 7.28 (d, J = 8.6 Hz, 2H), 5.27 (s, 2H), 3.99 (s, 3H), 3.95 (s, 3H).

Methyl 2-(4-hydroxyphenyl)-3-methyl-benzimidazole-5-carboxylate (1–3):

Methyl 2-(4-benzyloxyphenyl)-3-methyl-benzimidazole-5-carboxylate (**1–2**) (200 mg, 0.54

mmol) in dist. methanol (25 ml) was hydrogenated over a 10% Pd/C (100 mg) at 20 °C for 15 h. The solution was filtered through celite and concentrated to provide a white solid to give **1–3** in 66% yield. Rf = 0.15 (*n*-hexane/ethyl acetate = 1/1), ^{1}H NMR (600 MHz, DMSO-d_6): δ 10.1 (brs, 1H), 8.26 (d, J = 1.2 Hz, 1H), 7.91 (dd, J = 8.3, 1.4 Hz, 1H), 7.77 (t, J = 8.8 Hz, 3H), 7.00 (d, J = 8.6 Hz, 2H), 3.98 (s, 3H), 3.94 (s, 3H),

2-[4-[[3-(2,6-Dichlorophenyl)-5-isopropyl-isoxazol-4-yl]methoxy]phenyl]-3-methyl-benzimidazole-5-carboxylic acid (1):

To a solution of **1–3** (79 mg, 0.28 mmol) and 4-(chloromethyl)-3-(2,6-dichlorophenyl)-5-isopropyl-isoxazole (85 mg, 0.28 mmol) in DMF (4 ml), K_2CO_3 (120 mg, 0.84 mmol) was added at 20 °C. The reaction mixture was stirred at 70 °C for 15 h, followed by quenched with sat. NH_4Cl and extracted with ethyl acetate. The combined extracts were washed with H_2O and brine, dried over $MgSO_4$, and concentrated under the reduced pressure. The residue was purified by the silica gel column chromatography to give the ester form in 59% yield. Rf = 0.60 (*n*-hexane/ethyl acetate = 1/1). To a solution of the ester form (47 mg, 0.085 mmol) in methanol/THF (2 ml/1 ml), 1M NaOH (0.42 ml, 0.42 mmol) was added at 20 °C and the mixture was stirred at 20 °C for 15 h. The reaction mixture was neutralized with 1M HCl and evaporated down. The white solid product was appeared and filtrated to give 23 mg of **1** in 51% yield. Rf = 0.48 (CH_2Cl_2/methanol = 9/1), ^{1}H NMR (600 MHz, $CDCl_3$): δ 8.23 (s, 1H), 8.11 (d, J = 8.4 Hz, 1H), 7.90 (brs, 1H), 7.73 (d, J = 7.2 Hz, 2H), 7.42 (d, J = 8.4 Hz, 2H), 7.34 (dd, J = 15.0, 10.8 Hz, 1H), 6.96 (d, J = 7.8 Hz, 2H), 4.83 (s, 2H), 3.94 (s, 3H), 3.37 (sept., J = 7.2 Hz, 1H), 1.45 (d, J = 7.8 Hz, 6H)., HRMS: m/z found 536.11279 $[M+H]^+$. $C_{28}H_{24}Cl_2N_3O_4$ requires m/z 536.11439.

Synthetic protocol of 2:

Methyl 3-[(4-benzyloxybenzoyl)amino]-4-(methylamino)benzoate (2–1):

Methyl 3-amino-4-(methylamino)benzoate (0.49 g, 2.75 mmol) in DMF (5 ml) was added to a solution of 4-benzyloxybenzoic acid (0.62 g, 2.75 mmol) in DMF (4 ml) at 0 °C. Subsequently, HOAt (0.4 g, 3.0 mmol) and WSCI-HCl (0.58 g, 3.0 mmol) were added to the former solution at 0 °C and stirred at 20 °C for 15 h. The reaction mixture was quenched with sat. NH_4Cl and extracted with CH_2Cl_2. The combined extracts were washed with H_2O and brine, dried over $MgSO_4$, and concentrated under reduced pressure. The residue was purified by the silica gel column chromatography to give **2–1** in 58% yield. Rf = 0.65 (*n*-hexane/ethyl acetate = 1/1), ^{1}H NMR (600 MHz, DMSO-d_6): δ 9.55 (s, 1H), 8.03 (s, J = 8.6 Hz, 2H), 7.78 (brd, J = 8.6 Hz, 1H), 7.73 (brs, 1H), 7.53 (d, J = 7.4 Hz, 2H), 7.46 (t, J = 7.7 Hz, 2H), 7.40 (t, J = 7.3 Hz, 1H), 7.17 (d, J = 8.7 Hz, 2H), 6.70 (d, J = 8.6 Hz, 1H), 6.11 (brs, 1H), 5.27 (s, 2H), 3.81 (s, 3H), 2.82 (d, J = 4.8 Hz, 3H).

Methyl 2-(4-benzyloxyphenyl)-1-methyl-benzimidazole-5-carboxylate (2–2):

2–1 (520 mg, 1.33 mmol) was dissolved in CH_3COOH (16 ml) at 20 °C and stirred at 80 °C for 5 h. The reaction mixture was evaporated and quenched by addition of sat. $NaHCO_3$, followed by extracted with ethyl acetate. Organic layer was washed successively with H_2O and brine, dried over $MgSO_4$, and concentrated under reduced pressure. The residue was purified by the silica gel column chromatography to give **2–2** in 98% yield. Rf = 0.45 (*n*-hexane/ether = 1/2), ^{1}H NMR (600 MHz, DMSO-*d*$_6$): δ 8.30 (s, 1H), 7.97 (dd, J = 8.5, 1.5 Hz, 1H), 7.89 (d, J = 8.8 Hz, 2H), 7.77 (d, J = 8.5 Hz, 1H), 7.55 (d, J = 7.2 Hz, 2H), 7.48 (t, J = 7.4 Hz, 2H), 7.41 (t, J = 7.1 Hz, 1H), 7.27 (d, J = 8.8 Hz, 2H), 5.27 (s, 2H), 3.96 (s, 3H), 3.93 (s, 3H).

Methyl 2-(4-hydroxyphenyl)-1-methyl-benzimidazole-5-carboxylate (2–3):

Methyl 2-(4-benzyloxyphenyl)-1-methyl-benzimidazole-5-carboxylate (**2–2**) (230 mg, 0.61 mmol) in dist. methanol (30 ml) was hydrogenated over a 10% Pd/C (90 mg) at 20 °C for 15 h. The solution was filtered through celite and concentrated to provide a white solid to give **2–3** in 66% yield. Rf = 0.15 (*n*-hexane/ethyl acetate = 1/1), ^{1}H NMR (600 MHz, DMSO-*d*$_6$): δ 10.1 (brs, 1H), 8.28 (s, 1H), 7.95 (d, J = 8.5 Hz, 1H), 7.77 (d, J = 8.6 Hz, 2H), 7.75 (d, J = 8.6 Hz, 1H), 7.00 (d, J = 8.6 Hz, 2H), 3.94 (s, 3H), 3.93 (s, 3H).

2-[4-[[3-(2,6-Dichlorophenyl)-5-isopropyl-isoxazol-4-yl]methoxy]phenyl]-1-methyl-benzimidazole-5-carboxylic acid (2):

To a solution of **2–3** (79 mg, 0.28 mmol) and 4-(chloromethyl)-3-(2,6-dichlorophenyl)-5-isopropyl-isoxazole (85 mg, 0.28 mmol) in DMF (4 ml), K_2CO_3 (120 mg, 0.84 mmol) was added at 20 °C. The reaction mixture was stirred at 70 °C for 15 h, followed by quenched with sat. NH_4Cl and extracted with ethyl acetate. The combined extracts were washed with H_2O and brine, dried over $MgSO_4$, and concentrated under reduced pressure. The residue was purified by the silica gel column chromatography to give the ester form in 60% yield. Rf = 0.75 (*n*-hexane/ethyl acetate = 1/1). To a solution of the ester form (77 mg, 0.14 mmol) in methanol/THF (1 ml/2 ml), 1M NaOH (0.7 ml, 0.7 mmol) was added at 20 °C and the mixture was stirred at 20 °C for 15 h. The reaction mixture was neutralized with 1M HCl and evaporated down. The white solid product was appeared and filtrated to give 67 mg of **2** in 89% yield. Rf = 0.48 (CH_2Cl_2/methanol = 9/1), ^{1}H NMR (600 MHz, $CDCl_3$): δ 8.34 (s, 1H), 7.96 (d, J = 7.8 Hz, 1H), 7.86 (d, J = 9.0 Hz, 2H), 7.55 (d, J = 8.4 Hz, 1H), 7.40 (d, J = 7.2 Hz, 2H), 7.34 (dd, J = 9.0, 7.8 Hz, 1H), 6.98 (d, J = 8.4 Hz, 2H), 4.83 (s, 2H), 4.05 (s, 3H), 3.35 (sept., J = 7.2 Hz, 1H), 1.44 (d, J = 7.2 Hz, 6H)., HRMS: m/z found 536.11401 $[M+H]^+$. $C_{28}H_{24}Cl_2N_3O_4$ requires m/z 536.11439.

Synthetic protocol of 3:

Methyl 3-chloro-4-[[3-(2,6-dichlorophenyl)-5-isopropyl-isoxazol-4-yl]methoxy]benzoate

(3–1):

To a solution of methyl 3-chloro-4-hydroxy-benzoate (96 mg, 0.52 mmol) and 4-(bromomethyl)-3-(2,6-dichlorophenyl)-5-isopropyl-isoxazole (180 mg, 0.52 mmol) in DMF (3 ml), K_2CO_3 (140 mg, 1.0 mmol) was added at 20 °C. The reaction mixture was stirred at 70 °C for 15 h, followed by quenched with sat. NH_4Cl and extracted with ethyl acetate. The combined extracts were washed with H_2O and brine, dried over $MgSO_4$, and concentrated under reduced pressure. The residue was purified by the silica gel column chromatography to give **3-1** in 94% yield. Rf = 0.70 (*n*-hexane/ethyl acetate = 2/1), ^{1}H NMR (600 MHz, $CDCl_3$): δ 8.00 (d, J = 2.1 Hz, 1H), 7.83 (dd, J = 8.6, 2.2 Hz, 1H), 7.42 (brd, J = 8.3 Hz, 1H), 7.35 (d, J = 7.3 Hz, 1H), 7.34 (d, J = 7.3 Hz, 1H), 6.80 (d, J = 8.6 Hz, 1H), 4.87 (s, 2H), 3.88 (s, 3H), 3.39 (sept., J = 7.1 Hz, 1H), 1.43 (d, J = 7.0 Hz, 6H).

Methyl4-[[3-chloro-4-[[3-(2,6-dichlorophenyl)-5-isopropyl-isoxazol-4yl]methoxy]benzoyl] amino]-3-(methylamino)benzoate (3–2):

To a solution of **3–1** (220 mg, 0.48 mmol) in methanol (5 ml), 1M NaOH (0.96 ml, 0.96 mmol) was added at 25 °C and the mixture was stirred at 25 °C. for 15 h The reaction mixture was neutralized with 1M HCl and evaporated down. The white solid product was appeared and filtrated (208 mg). The white product (100 mg, 0.23 mmol) and methyl 4-amino-3-(methylamino)benzoate (41 mg, 0.23 mmol) were dissolved in 2 ml of DMF. Subsequently, HOAt (33 mg, 0.25 mmol) and WSCI-HCl (48 mg, 0.25 mmol) were added to the former solution at 0 °C and stirred at 25 °C for 15h. The reaction mixture was quenched with sat. NH_4Cl and extracted with ethyl acetate. The combined extracts were washed with H_2O and brine, dried over $MgSO_4$, and concentrated under reduced pressure. The residue was purified by the silica gel column chromatography to give **3–2** in 50% yield. Rf = 0.75 (*n*-hexane/ethyl acetate = 1/1), ^{1}H NMR (600 MHz, $CDCl_3$): δ 7.88 (d, J = 2.2 Hz, 1H), 7.77 (brs, 1H), 7.71 (brd, J = 6.7 Hz, 1H), 7.55 (brs, 2H), 7.52 (s, 1H), 7.43 (dd, J = 8.0, 0.84 Hz, 1H), 7.37 (d, J = 7.3 Hz, 1H), 7.35 (d, J = 7.3 Hz, 1H), 6.87 (d, J = 8.7 Hz, 1H), 4.90 (s, 2H), 3.92 (brs, 4H), 3.41 (sept., J = 7.0 Hz, 1H), 2.91 (s, 3H), 1.45 (d, J = 7.0 Hz, 6H).

Methyl 2-[3-chloro-4-[[3-(2,6-dichlorophenyl)-5-isopropyl-isoxazol-4-yl]methoxy]phenyl]-3-methyl-benzimidazole-5-carboxylate (3–3):

3–2 (51 mg, 0.085 mmol) was dissolved in CH_3COOH (2 ml) at 20 °C and stirred at 80 °C for 2 h. The reaction mixture was evaporated and quenched by adding of sat. $NaHCO_3$. The mixture was extracted with ethyl acetate, and organic layer was washed successively with H_2O and brine, dried over $MgSO_4$, and concentrated under reduced pressure. The residue was purified by the silica gel column chromatography to give **3–3** in 93% yield. Rf = 0.70 (*n*-hexane/ethyl acetate = 1/1), ^{1}H NMR (600 MHz, $CDCl_3$): δ 8.15 (brs, 1H), 8.02 (dd, J = 8.4, 1.6 Hz, 1H), 7.79-7.78 (m, 2H), 7.60 (dd, J = 8.5, 2.2 Hz, 1H), 7.44 (dd, J = 8.1, 0.90 Hz, 1H), 7.37 (d,

J = 7.3 Hz, 1H), 7.35 (d, J = 7.3 Hz, 1H), 6.94 (d, J = 8.6 Hz, 1H), 4.91 (s, 2H), 3.97 (s, 3H), 3.91 (s, 3H), 3.43 (sept., J = 7.0 Hz, 1H), 1.46 (d, J = 7.0 Hz, 6H).

2-[3-Chloro-4-[[3-(2,6-dichlorophenyl)-5-isopropyl-isoxazol-4-yl]methoxy]phenyl]-3-methyl-benzimidazole-5-carboxylic acid (3):

To a solution of **3–3** (46 mg, 0.079 mmol) in methanol/THF (1 ml/1 ml), 1M NaOH (0.23 ml, 0.23 mmol) was added at 25 °C and the mixture was stirred at 25 °C for 15 h. The reaction mixture was neutralized with 1M HCl and evaporated down. The white solid product was appeared and filtrated to give 36 mg of **3** in 81% yield. ^{1}H NMR (600 MHz, $CDCl_3$): δ 8.22 (d, J = 1.2 Hz, 1H), 8.09 (dd, J = 9.0, 1.2 Hz, 1H), 7.84 (d, J = 7.8 Hz, 1H), 7.81 (d, J = 2.4 Hz, 1H), 7.62 (dd, J = 7.8, 2.4 Hz, 1H), 7.44 (d, J = 7.8 Hz, 2H), 7.36 (dd, J = 7.8, 6.6 Hz, 1H), 6.95 (d, J = 8.4 Hz, 1H), 4.92 (s, 2H), 3.93 (s, 3H), 3.43 (sept., J = 6.6 Hz, 1H), 1.46 (d, J = 6.6 Hz, 6H)., HRMS: m/z found 570.07551 $[M+H]^+$. $C_{28}H_{23}Cl_3N_3O_4$ requires m/z 570.07541.

Synthetic protocol of 4:

Methyl 2-chloro-4-[[3-(2,6-dichlorophenyl)-5-isopropyl-isoxazol-4-yl]methoxy]benzoate (4–1):

To a solution of methyl 2-chloro-4-hydroxy-benzoate (120 mg, 0.65 mmol) and 4-(bromomethyl)-3-(2,6-dichlorophenyl)-5-isopropyl-isoxazole (250 mg, 0.72 mmol) in DMF (3 ml), K_2CO_3 (200 mg, 1.4 mmol) was added at 20 °C. The reaction mixture was stirred at 70 °C for 15 h, followed by quenched with sat. NH_4Cl and extracted with ethyl acetate. The combined extracts were washed with H_2O and brine, dried over $MgSO_4$, and concentrated under reduced pressure. The residue was purified by the silica gel column chromatography to give **4–1** in 80% yield. Rf = 0.75 (*n*-hexane/ethyl acetate = 2/1), ^{1}H NMR (600 MHz, $CDCl_3$): δ 7.80 (d, J = 8.8 Hz, 1H), 7.41 (d, J = 8.3 Hz, 1H), 7.33 (t, J = 7.4 Hz, 2H), 6.83 (brs, 1H), 6.69 (brd, J = 8.8 Hz, 1H), 4.76 (s, 2H), 3.88 (s, 3H), 3.32 (sept., J = 7.0 Hz, 1H), 1.43 (d, J = 7.0 Hz, 6H).

Methyl 4-[[2-chloro-4-[[3-(2,6-dichlorophenyl)-5-isopropyl-isoxazol-4-yl]methoxy]benzoyl]amino]-3-(methylamino)benzoate (4–2):

To a solution of **4–1** (236 mg, 0.52 mmol) in methanol (6 ml), 1M NaOH (1.0 ml, 1.0 mmol) was added at 25 °C. The mixture was stirred at 25 °C for 15 h and then neutralized with 1M HCl and evaporated down. The white solid product was appeared and filtrated (248 mg). The white product (120 mg, 0.27 mmol) and methyl 4-amino-3-(methylamino)benzoate (48 mg, 0.27 mmol) were dissolved in 2 ml of DMF. Subsequently, HOAt (39 mg, 0.3 mmol) and WSCI-HCl (58 mg, 0.3 mmol) were added to the former solution at 0 °C and stirred at 25 °C for 15 h. The reaction mixture was quenched with sat. NH_4Cl and extracted with ethyl acetate.

The combined extracts were washed with H_2O and brine, dried over $MgSO_4$, and concentrated under reduced pressure. The residue was purified by the silica gel column chromatography to give **4–2** in 40% yield. Rf = 0.65 (*n*-hexane/ethyl acetate = 1/1), ^1H NMR (600 MHz, $CDCl_3$): δ 8.01 (brs, 1H), 7.82 (d, J = 8.8 Hz, 1H), 7.62 (d, J = 8.1 Hz, 1H), 7.53 (d, J = 8.5 Hz, 1H), 7.49 (s, 1H), 7.42 (d, J = 7.4 Hz, 1H), 7.36 (d, J = 7.3 Hz, 1H), 7.34 (d, J = 7.3 Hz, 1H), 6.84 (brs, 1H), 6.79 (brd, J = 8.8 Hz, 1H), 4.79 (s, 2H), 3.92 (s, 4H), 3.33 (sept., J = 6.8 Hz, 1H), 2.92 (s, 3H), 1.45 (d, J = 7.0 Hz, 6H).

Methyl 2-[2-chloro-4-[[3-(2,6-dichlorophenyl)-5-isopropyl-isoxazol-4-yl]methoxy]phenyl]-3-methyl-benzimidazole-5-carboxylate (4–3)

4–2 (63 mg, 0.1 mmol) was dissolved in CH_3COOH (2 ml) at 20 °C and stirred at 80 °C for 2 h. The reaction mixture was evaporated and quenched by addition of sat. $NaHCO_3$. The mixture was extracted with ethyl acetate, and organic layer was washed successively with H_2O and brine, dried over $MgSO_4$, and concentrated under reduced pressure. The residue was purified by the silica gel column chromatography to give **4–3** in 85% yield. Rf = 0.70 (*n*-hexane/ethyl acetate = 1/1), ^1H NMR (600 MHz, $CDCl_3$): δ 8.17 (brs, 1H), 8.03 (brd, J = 8.5 Hz, 1H), 7.81 (d, J = 8.5 Hz, 1H), 7.43 (dd, J = 8.3, 1.9 Hz, 2H), 7.37 (d, J = 7.5 Hz, 1H), 7.35 (d, J = 8.8 Hz, 1H), 6.93 (d, J = 2.4 Hz, 1H), 6.82 (dd, J = 8.5, 2.5 Hz, 1H), 4.82 (s, 2H), 3.98 (s, 3H), 3.70 (s, 3H), 3.36 (sept., J = 7.0 Hz, 1H), 1.46 (d, J = 7.1 Hz, 6H).

2-[2-Chloro-4-[[3-(2,6-dichlorophenyl)-5-isopropyl-isoxazol-4-yl]methoxy]phenyl]-3-methyl-benzimidazole-5-carboxylic acid (4):

To a solution of **4–3** (51 mg, 0.087 mmol) in methanol/THF (1 ml/1 ml), 1M NaOH (0.26 ml, 0.26 mmol) was added at 25 °C and the mixture was stirred at 25 °C for 15 h. The reaction mixture was neutralized with 1M HCl and evaporated down. The white solid product was appeared and filtrated to give 42 mg of **4** in 85% yield. ^1H NMR (600 MHz, $CDCl_3$): δ 8.29 (brs, 1H), 8.17 (d, J = 7.8 Hz, 1H), 8.04 (brd, J = 7.8 Hz, 1H), 7.63 (d, J = 6.6 Hz, 1H), 7.44 (d, J = 8.4 Hz, 2H), 7.37 (dd, J = 9.0, 7.2 Hz, 1H), 6.98 (d, J = 2.4 Hz, 1H), 6.91 (brd, J = 7.2 Hz, 1H), 4.83 (s, 2H), 3.82 (s, 3H), 3.36 (sept., J = 6.6 Hz, 1H), 1.46 (d, J = 6.6 Hz, 6H)., HRMS: m/z found 570.07508 $[M+H]^+$. $C_{28}H_{23}Cl_3N_3O_4$ requires m/z 570.07541.

Table S1. Purity of novel synthetic FXR agonists

FXR agonist	Purity (%)	Retention time (min)
**1**	97.6	18.603
**2**	97.1	18.595
**3**	92.4	20.484
**4**	97.7	19.790

© 2019 by the authors. Licensee MDPI, Basel, Switzerland. This article is an open access article distributed under the terms and conditions of the Creative Commons Attribution (CC BY) license (http://creativecommons.org/licenses/by/4.0/).
